# A novel digital biomarker of sarcopenia in frail elderly: New combination of gait parameters under dual-task walking

**DOI:** 10.3389/fnagi.2023.1087318

**Published:** 2023-02-20

**Authors:** Zheping Zhou, Yulun Huang, Jing Wang, Huan Su, Haiying Tang, Yueju Wang

**Affiliations:** ^1^Department of Geratology, The First Affiliated Hospital of Soochow University, Suzhou, China; ^2^Department of Neurosurgery, Dushu Lake Hospital Affiliated to Soochow University, Suzhou, China

**Keywords:** frailty, sarcopenia, gait, dual-task walking, digital biomarker

## Abstract

**Background:**

Frailty caused by deterioration in multiple physiological systems has led to a significant increase in adverse events such as falls, disability, and death in frail older people. Similar to frailty, sarcopenia, defined as loss of skeletal muscle mass and strength, is tightly related to mobility disorders, falls, and fractures. With population aging, co-occurrences of frailty and sarcopenia are increasingly common in the elderly, which are more deleterious for the health and independence of older adults. But the high similarity and overlap between the frailty and sarcopenia increase the difficulty of early recognition of frailty with sarcopenia. The purpose of this study is to use detailed gait assessment to determine the more convenient and sensitive digital biomarker of sarcopenia in the frail population.

**Methods:**

Ninety-five frail elderly people (age = 86 ± 7 years old, BMI, and body mass index = 23.21 ± 3.40 kg/m^2^) were screened out by the evaluation of Fried criteria. Then, 41 participants (46%) were identified with sarcopenia, and 51 participants (54%) were identified without sarcopenia. Using a validated wearable platform, participants’ gait performance was evaluated under single-task and dual-task (DT). Participants walked back and forth on the 7-m-long trail for 2 min at a habitual speed. Gait parameters of interest include cadence, gait cycle duration, step duration, gait speed, variability of gait speed, stride length, turn duration, and steps in turn.

**Results:**

Our results showed that compared with the frail elderly without sarcopenia, the gait performance of the sarcopenic group in single-task and dual-task walking was worse. Overall, the parameters with high performance were the gait speed (DT) (OR 0.914; 95% CI 0.868–0.962) and turn duration (DT) (OR 7.907; 95% CI 2.401–26.039) under dual-task conditions, and the AUC in distinguishing between frail older adults with and without sarcopenia was 0.688 and 0.736, respectively. Turn duration in dual-task testing had larger observed effect than gait speed to identify sarcopenia in the frail population, this result remained significant even after controlling for potential confounds. When gait speed (DT) and turn duration (DT) were combined in the model, AUC increased from 0.688 to 0.763.

**Conclusion:**

This study shows that gait speed and turn duration under dual-task are good predictors of sarcopenia in frail elderly, and turn duration (DT) has a better predictive ability. The gait speed (DT) combined with turn duration (DT) is a potential gait digital Biomarker of sarcopenia in the frail elderly. Dual-task gait assessment and detailed gait indexes provide important value for identification of sarcopenia in frail elderly people.

## 1. Introduction

As a clinically relevant syndrome, frailty is defined by Fried et al. as a series of syndromes that result in an increased vulnerability to stressors, related to the deterioration in multiple physiological systems, typically presenting with slow step velocity, decreased activity, weight loss, fatigue, and muscle weakness (Fried et al., 2001). This could increase the risk of adverse events in old adults, including functional decline, falls, and high hospitalization rates (Brummel et al., 2017). Meanwhile, frailty was independently associated with disability and death (Fried et al., 2001; Wewerka et al., 2015; Sáez de Asteasu et al., 2019). And a growing number of studies have revealed that gait speed is one of the strongest indexes to predict adverse outcomes and the most useful indicator for the identification of frailty (Montero-Odasso et al., 2005; Rothman et al., 2008; Binotto et al., 2018).

Recent studies revealed that there were a large number of sarcopenic patients caused by age-related decline in muscle mass and strength in the frail older population (Roberts et al., 2021). Sarcopenia is defined as loss of skeletal muscle mass and strength (Cruz-Jentoft et al., 2010). Similar to frailty, sarcopenia is related to mobility disorders, falls and fractures, and severe reductions in their independence in activities of daily living (Liu et al., 2021). Frailty and sarcopenia share high symptomatic similarity and diagnostic overlap and thus cannot easily be distinguished (Fried et al., 2001; Cederholm, 2015; Mijnarends et al., 2015). As life spans lengthen, frailty and sarcopenia of aging are becoming more common, significantly affecting function and quality of life of elderly (Inoue et al., 2018). There has been a concomitant increase in frailty with sarcopenia, which can be more harmful to elderly’s health and quality of life. This condition requires early detection and careful treatment (Carmeli, 2017).

When sarcopenia occurs in the frail elderly population, the risks of falls, disability, and death will be substantially elevated in these individuals (Pfortmueller et al., 2014; Marques and Queirós, 2018; Thompson et al., 2021). A meta-analysis study revealed that there was a high prevalence of overlap between frailty and sarcopenia in hospitalized older adults, the combination of these conditions probably synergistically impairs outcome, leading to its adverse prognosis, including mobility disorders, falls, and fractures (Ligthart-Melis et al., 2020). A latest 10-year follow-up study demonstrated that a combination model of frailty and sarcopenia was more predictive of mortality than either condition alone. Patients with both frailty and sarcopenia were at nearly double risk of mortality than those with just one of these two diseases, and were over three times at risk of mortality than those neither frail nor sarcopenic. Individuals identified as frail would benefit from screening and assessment for sarcopenia (Thompson et al., 2021). Moreover, individuals with sarcopenia and frailty showed the strongest associations with cardiovascular disease and respiratory disease incidence, and mortality for all-cause and respiratory disease and cancer, and those with both sarcopenia and frailty had the highest mortality rate (Petermann-Rocha et al., 2021). Consequently, early recognition of sarcopenia in the frail elderly would be of particular significance, the aim is to start prevention as early as possible through exercise, nutrition, supplementation, and medical intervention. This approach could effectively decrease fall rates, disability rates, and mortality.

Stride speed has played an important role in the diagnosis of frailty and sarcopenia (Cruz-Jentoft et al., 2010). However, Gait encompasses a broad array of quantifiable parameters, such as cadence, gait speed, stride length, gait variability, and so on, and they reflect various aspects of gait (Verlinden et al., 2013). Studies focusing on the comprehensive gait assessment of the frail population and sarcopenic patients are still few. The gait characteristic of sarcopenic patients in the frail elderly has not been covered in literature so far. Due to the difficulty in the measurement of skeletal muscle, sarcopenia in the frail elderly is often not detected on time, sarcopenia comorbid with frailty will always result in a much worse prognosis, significant loss of quality of life, and can even lead to mortality (Petermann-Rocha et al., 2021). We can see that most of the older adults move slowly and show a significantly decline in daily physical activity. Recent work in this field suggested that there was a considerable overlap between frailty and sarcopenia in older adults (Ligthart-Melis et al., 2020; Faxén-Irving et al., 2021; AlMohaisen et al., 2022). However, diagnosis of sarcopenia has received little attention in these frail older adults and good and timely intervention therefore cannot be guaranteed. This special population needs much weighted attention, as they are worse and more hidden.

Few studies have focused on dual-task (DT) gait testing (walking while simultaneously performing a cognitive task) as a means to predict the onset of frailty or sarcopenia (Verghese et al., 2012; Thiede et al., 2016). Past research showed that gait changes would become more marked in the dual-task testing, the measurement of step speed under dual-task testing would enable researchers to more effectively distinguish between older adults with and without frailty (Guedes et al., 2014; Montero-Odasso et al., 2015). However, the researches of gait in dual-task conditions specific to frail older adults or sarcopenic patients are still inadequate. Combining Single- and dual-task gait analysis can reveal the feature of gait more comprehensively. Thus, we introduce the gait parameters within the dual-task trials for a deeper and more comprehensive understanding of gait characteristics in the frail elderly with sarcopenia.

No other study has taken a gait measurement technique to early analysis of sarcopenia in frail older adults to the best of our knowledge. Therefore, this study aims to examine the gait parameters of frail elderly population and its association with sarcopenia, using a gait assessment protocol including single- and dual-task gait testing to develop the model of gait characteristics for the early screening of frail older adults with sarcopenia.

## 2. Methods

### 2.1. Study design and participants

Five hundred and six randomly selected people aged 75 years and over from three different care homes were invited to participate in the cross-sectional study organized by First Affiliated Hospital of Soochow University. Three hundred and four patients were excluded from the initial sample for neurological diseases that can cause severe walking disability, lower limb musculoskeletal disorders which may influence gait performance (such as severe arthritis, lower extremity trauma, or surgery), and severe psychiatric disorders (such as major depression or schizophrenia). Two hundred and two participants were tested with the cognition scale, gait test, and the bioelectric impedance method (BIA). We again excluded the following patients: (1) patients who are incapable of walking without assistance from another person, (2) patients who cannot provide informed consent, (3) patients who unable to complete the dual-task gait testing, and (4) missing Cognitive and physical functioning data during the tests. Ultimately, according to comprehensive test results and frailty phenotype criteria, 95 participants diagnosed with frailty were included in the study. Ethical approval was obtained from the Ethical Committee of the First Affiliated Hospital of Soochow University.

### 2.2. Medical and cognitive assessments

Sociodemographic characteristics and medical history (osteoporotic, hypertension, diabetes mellitus, cerebrovascular disease, coronary heart disease, and anxiety/depression) were collected using standardized questionnaires during face-to-face interviews. Recent laboratory findings were collected by study doctors.

The MoCA-Beijing used in current study is one of the five Chinese versions of MoCA, and has been translated and used in previous studies with clinical populations (Yu et al., 2012). The items that are used to examine the seven cognitive domains (i.e., visuospatial/executive function, naming, attention, abstraction, language, delayed memory, and orientation) are translated from the original English version literally. The medical and cognitive assessments were performed by trained neuropsychologists.

### 2.3. Frailty assessment

We used the frailty phenotype criteria (Fried et al., 2001). (1) Unintentional weight loss ≥4.6 kg or ≥ 5% in the last year. (2) Weakness as measured by grip strength, using a KYTO^®^ hand dynamometer, in the lowest 20%, adjusted for gender and body mass index. (3) Poor energy and endurance, as indicated by self-reported exhaustion determined by two questions from the Center of Epidemiologic Studies Depression Scale. (4) Slowness, measured as the time taken to walk 4.57 m, within the lowest 20th percentile and adjusted for gender and height. (5) Low physical activity level, according to the patient self-reported the outdoor activity time of the past week, men <2.5 h and women <2 h. To construct the frailty phenotype variable, participants had to have valid values in at least 3 of the 5 criteria. Subjects were considered frail if three or more criteria were present.

### 2.4. Diagnosis of sarcopenia

The diagnosis of sarcopenia is based on the consensus report of the Asian Working Group for Sarcopenia (AWGS), including low muscle strength, low muscle mass, and/or low physical performance (Chen et al., 2014). The maximum grip strength was measured twice for each hand using a KYTO^®^ hand dynamometer. Low muscle strength was defined as grip strength <26 kg in men and <18 kg in women. The skeletal muscle mass was measured using Bioelectrical impedance analysis (InBody720, Seoul, Korea). Low muscle mass was defined as an SMI (skeletal muscle mass/height2) of <7.0 kg/m2 in men and < 5.4 kg/m^2^ in women. Low physical performance was defined as a gait speed of ≤0.8 m/s, measured by APDM Movement Monitoring inertial sensor system (APDM Inc., Portland, OR, United States).

### 2.5. Gait assessments

**Figure 1:**
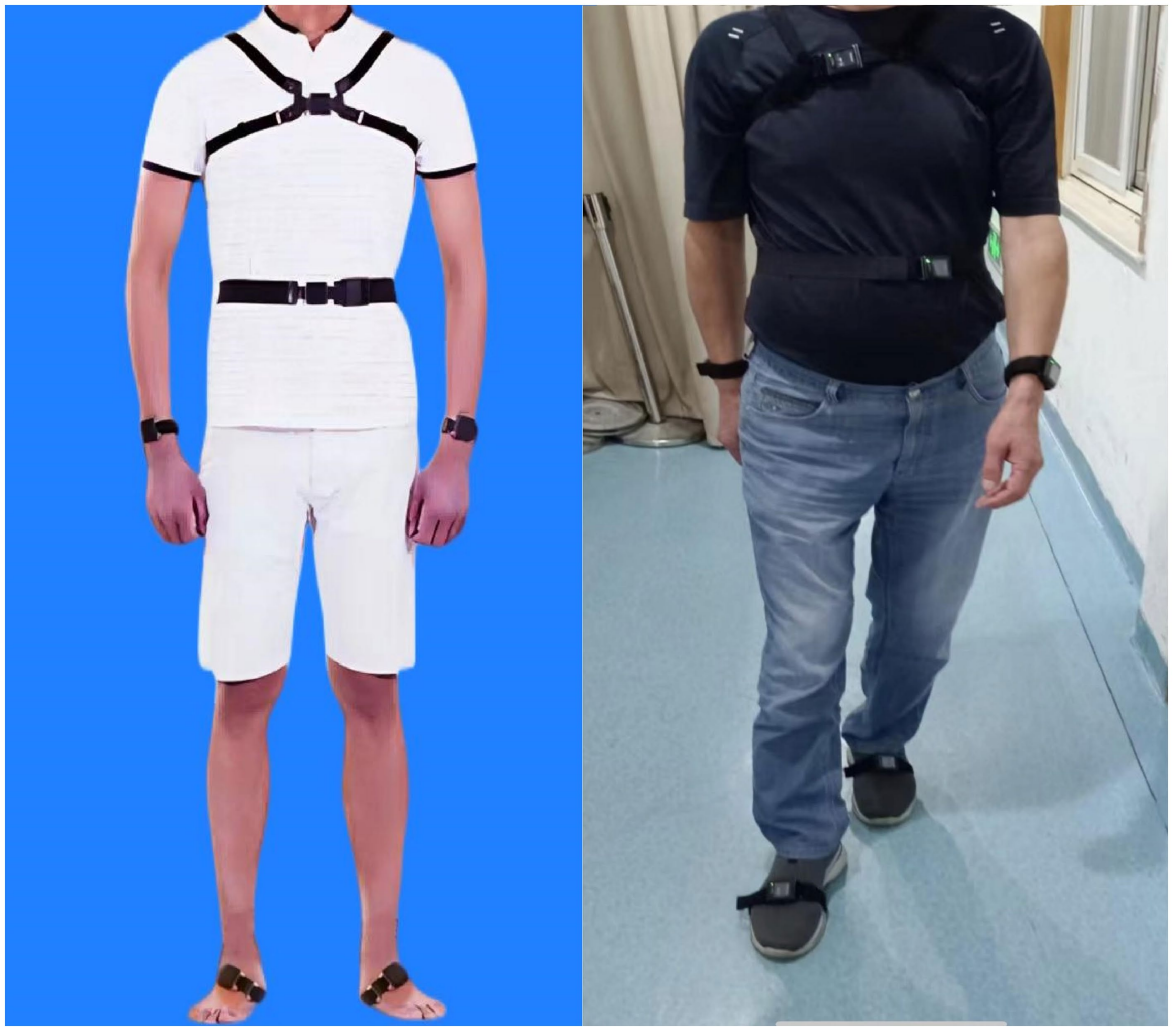
The wireless APDM Movement Monitoring inertial sensor system, six opal inertial sensors (APDM Inc.) were worn on the wrists, ankles, sacrum, and chest during walking.

Gait parameters within single- and dual-tasks were assessed using a wireless APDM Movement Monitoring inertial sensor system (APDM Inc., Portland, OR, United States) that provides data to assess both spatial and temporal gait parameters. Six opal inertial sensors (APDM Inc.) were worn on the wrists, ankles, sacrum, and chest during walking; Mobility Lab software (APDM, Inc.) analyzed gait characteristics automatically (Mancini et al., 2011; see Figure 1). The subjects were asked to walk back and forth for 2 min on a 7-m-long straight sidewalk (with colored tape marking the start and end points) at their usual speed, wearing comfortable clothing and shoes, without the use of any mobility aids. Prior to the trials, participants were giving standardized instructions and a visual demonstration. The verbal instructions were given: “(1) When you hear the first sound, start walking at a natural and comfortable pace. (2) When you hear the second sound, stop walking.” In the dual task test, participants walked at their usual speed while doing the following cognitive tasks aloud: (1) count backward from 100, (2) subtract consecutive sevens from 100, and (3) name animals. The protocol has established reliability in other studies (Montero-Odasso et al., 2009). Participants were asked to perform three single-task trials and three dual-task trials, the mean of the three trials was used in the analysis. To balance and reduce the effects of learning and fatigue, only one test was performed in each condition, 5-min rest provided between each trial. The order of single- and double-tasks was random. All procedures were performed by the same professional gerontologist, who stood by to observe the participants for protection.

We selected 8 gait parameters of interest in each condition (single- and dual-task condition) for further analysis: in Table 1, we provide descriptions of these parameters.

**Table 1:** Descriptions of gait parameters.

Parameter	Definition
Cadence	The number of steps/min
Gait cycle duration	The time elapsed between the last contact of the current footfall to the first contact of the next footfall on the same foot in seconds
Step duration	The time elapsed between the first contact of one foot and the first contact of the opposite foot
Gait speed	The velocity in centimeters/s
Gait speed std	The standard deviation in the gait speed, represents speed variability
Stride length	The distance between the heel points of two consecutive footprints of the same foot on the line of progression in centimeters
Turn duration	The turning time was defined as the time between the last contact of the second foot before the first turn foot and the first contact of the second foot with a normal angle coming out of the turn
Steps in turn	The number of steps used within the turning time

### 2.6. Statistical analysis

Data were analyzed using SPSS23.0, the Skewness/Kurtosis test was used to test for normal distributions of continuous variables. Groups’ frailty and frailty with sarcopenia were compared using the *t*-test for quantitative variables, variables were described using means and standard deviations if the data were normally distributed. When normality was not fulfilled, nonparametric tests (Mann–Whitney *U*-test) were used, non-normally distributed variables were expressed as medians and interquartile range. Chi-square test was used for the comparison of rate. Variables with statistical significance in the univariate analysis were investigated by multivariate logistic regression analysis. The odds ratios and 95% confidence intervals before and after controlling simultaneously for potential confounders (sex, BMI, and history of osteoporosis) were calculated. Additionally, we analyzed associations between specific gait parameters and continuous variables (SMI, grip strength, and FREID scores) using Pearson and Spearman’s correlations. Finally, ROC curves were constructed to determine the best association between gait parameters and frailty with sarcopenia.

## 3. Results

### 3.1. Participant characteristics

Table 2 shows the demographic characteristics of 95 participants with frailty classified by state of sarcopenia. According to the Fried criteria and AWGS criteria, a total of 44 (46.3%) participants were classified as frailty with sarcopenia. The frail group and the frail group with sarcopenia did not differ significantly with respect to age (87.522 ± 6.296 vs. 85.176 ± 7.320, *p* > 0.05). The sarcopenic group had a higher proportion of male cases (63.64% vs. 36.36%, *p* < 0.05) and lower BMI and SMI when compared with non-sarcopenic patients (*p* < 0.05). Significantly, sarcopenia patients were more likely to have greater severity of frailty presented with higher FREID scores (5(5,5) vs. 4(3,4), *p* < 0.05), slower stride velocity (*p* < 0.01), and longer 4.57 m walking time (*p* < 0.05), but there were no differences in grip strength among the groups (*p* > 0.05).

**Table 2:** Sociodemographic and medical characteristics.

Variable	Frailty	Frailty with sarcopenia	*t*/*z*/*χ*^2^	Value of *p*
	*N* = 51	*N* = 44		
Age (years)	85.176 ± 7.320	87.522 ± 6.296	−1.661	0.100
BMI (kg/m^2^)	24.294 ± 3.313	21.959 ± 3.079	3.539	0.001
SMI (kg/m^2^)	7.200 ± 1.011	5.780 ± 5.555	7.714	<0.001
Cr (μmol/L)	76.467 ± 27.512	77.066 ± 15.734	−0.128	0.899
UA (μmol/L)	346.455 ± 107.029	325.614 ± 98.982	0.978	0.329
Alb (g/L)	40.931 ± 5.904	42.602 ± 7.825	−1.183	0.240
TC (mmol/L)	3.94 ± 0.955	3.827 ± 1.264	0.496	0.621
TG (mmol/L)	1.490 ± 0.735	1.333 ± 0.697	1.056	0.294
HDLC (mmol/L)	1.230 ± 0.339	1.202 ± 0.327	0.396	0.693
LDLC (mmol/L)	2.462 ± 0.613	2.266 ± 0.675	1.484	0.141
HBDH (U/L)	150.582 ± 35.454	150.932 ± 41.879	−0.044	0.965
Gait speed (cm/s)	51.333 ± 17.765	39.863 ± 11.027	3.558	0.001
MoCA	18.157 ± 4.483	17.295 ± 6.021	0.797	0.427
M visuospatial/executive function	2.156 ± 1.433	2.205 ± 1.636	−0.151	0.880
M naming	2.803 ± 0.633	2.659 ± 0.568	1.165	0.246
M attention	4.471 ± 1.447	4,136 ± 1.607	1.066	0.289
M language	1.667 ± 0.887	1.523 ± 1.067	0.718	0.475
M abstraction	1.216 ± 0.672	1.295 ± 0.733	−0.553	0.582
M delayed memory	0.882 ± 1.227	1.182 ± 1.419	−1.103	0.273
M orientation	4.980 ± 1.225	4.227 ± 1.626	2.569	0.012
Clock drawing task	2.118 ± 1.321	2.045 ± 1.396	0.259	0.797
FREID scores	4 (3,4)	5 (5,5)	−8.377	<0.001
4.57 m walking time(s)	9.327 (7.371,11.718)	12.189 (9.235,14.281)	−2.905	0.004
Grip strength(kg)	15.5 (11.3,22.7)	14.9 (9.95,21.7)	−0.795	0.472
Gender				
Female, *n* (%)	29 (56.86)	16 (36.36)	3.981	0.046
Male, *n* (%)	22 (43.14)	28 (63.64)		
Osteoporotic, *n* (%)				
No	45 (88.24)	30 (68.18)	5.715	0.017
Yes	6 (11.76)	14 (31.82)		
Hypertension, *n* (%)				
No	18 (35.29)	17 (38.64)	0.113	0.736
Yes	33 (64.71)	27 (61.36)		
Diabetes mellitus, *n* (%)				
No	35 (68.63)	35 (79.55)	1.452	0.228
Yes	16 (31.37)	9 (20.45)		
Cerebrovascular disease, *n* (%)				
No	31 (60.78)	31 (70.45)	0.974	0.324
Yes	20 (39.22)	13 (29.55)		
Coronary heart disease, *n* (%)				
No	40 (78.43)	35 (79.55)	0.018	0.894
Yes	11 (21.57)	9 (20.45)		
Anxiety/Depression, *n* (%)				
No	42 (82.35)	37 (84.09)	0.051	0.821
Yes	9 (17.65)	7 (15.91)		

BMI, Body mass index; SMI, skeletal muscle mass index; Cr, creatinine; UA, uric acid; Alb, albumin; TC, total cholesterol; TG, triglyceride; HDLC, high-density lipoprotein cholesterol; LDLC, low-density lipoprotein cholesterol; HBDH, hydroxybutyrate dehydrogenase.

Fewer patients without sarcopenias had a history of osteoporosis (11.76%) compared to 31.82% of those diagnosed with sarcopenia (*p* < 0.05). No significant differences were found in MoCA scores between the groups adjusted for age, sex, and education (*p* > 0.05), but sarcopenic patients showed lower orienting ability than non-sarcopenic patients (4.227 ± 1.626 vs. 4.980 ± 1.225, *p* < 0.05). The recent results of blood biochemical (creatinine, uric acid, albumin, total cholesterol, triglyceride, high-density lipoprotein cholesterol, low-density lipoprotein cholesterol, and hydroxybutyrate dehydrogenase) showed no significant difference between the two groups (*p* > 0.05).

### 3.2. Gait performance

As shown in Table 3. Frail patients with sarcopenia had overall a poor performance, both in single- and dual-task. For the single-gait test, the sarcopenic group showed slower stride velocity (39.863 ± 11.027 cm/s vs. 51.333 ± 17.765 cm/s, *p* < 0.01) and shorter stride length (47.977 ± 13.305 cm vs. 62.254 ± 21.592 cm, *p* < 0.01). For the dual-task tests, the sarcopenic group showed slower gait speed (29.068 ± 9.219 cm/s vs. 37.313 ± 13.979 cm/s, *p* < 0.01), lower gait speed std. (5.931 ± 2.773 cm/s vs. 7.431 ± 3.556 cm/s, *p* < 0.05), shorter stride length (37.977 ± 11.475 cm vs. 49.235 ± 18.504 cm, *p* < 0.01), and longer turn duration (3.253 ± 0.537 s vs. 2.847 ± 0.509 s, *p* < 0.01) compared to the non-sarcopenic group.

**Table 3:** Gait performance for groups with and without sarcopenia.

Variable	Frailty	Frailty with sarcopenia	*t*	Value of *p*
	*N* = 51	*N* = 44		
Single-task gait				
Cadence (steps/min)	99.814 ± 13.900	100.755 ± 12.262	−0.347	0.729
Gait cycle duration (s)	1.230 ± 0.179	1.216 ± 1.171	0.404	0.688
Step duration (s)	0.612 ± 0.093	0.609 ± 0.081	0.145	0.885
Gait speed (cm/s)	51.333 ± 17.765	39.863 ± 11.027	3.558	0.003
Gait speed std. (cm/s)	6.745 ± 2.614	7.182 ± 2.990	−0.760	0.775
Stride length (cm)	62.254 ± 21.592	47.977 ± 13.305	3.805	<0.001
Turn duration (s)	2.940 ± 0.430	3.049 ± 0.524	−1.107	0.271
Steps in turn (*n*)	4.704 ± 1.185	4.978 ± 1.314	−1.064	0.290
Dual-task gait				
Cadence (steps/min) [DT]	91.525 ± 16.144	93.897 ± 15.994	−0.717	0.475
Gait Cycle Duration (s) [DT]	1.363 ± 0.246	1.324 ± 0.221	0.811	0.419
Step Duration (s) [DT]	0.689 ± 0.139	0.664 ± 0.116	0.919	0.360
Gait Speed (cm/s) [DT]	37.313 ± 13.979	29.068 ± 9.219	3.335	0.001
Gait Speed std. (cm/s) [DT]	7.431 ± 3.556	5.931 ± 2.773	2.264	0.013
Stride Length (cm) [DT]	49.235 ± 18.504	37.977 ± 11.475	3.496	0.001
Turn Duration (s) [DT]	2.847 ± 0.509	3.253 ± 0.537	−3.774	<0.001
Steps in Turn (*n*) [DT]	4.281 ± 1.231	4.930 ± 1.282	−2.511	0.993

### 3.3. Multiple logistics regression

As shown in Tables 4, 5, after adjusting for confounders (sex, BMI, history of osteoporosis), logistic regression analysis was used for data analysis. As gait parameters in single-task gait parameters indicated, gait speed and stride length were suited for the logistic regression model, each 1 cm/s increase in gait speed decreased the risk of disease by 7.8% (OR 0.922; 95% CI 0.881–0.965), each 1 cm increase in stride length decreased the risk of disease by 6.1% (OR 0.939; 95% CI 0.906–0.972). Gait speed (DT), gait speed std. (DT), stride length (DT), and turn duration (DT) were suited for the logistic regression model under the dual-task condition, each 1 cm/s increase in gait speed (DT) decreased the risk of disease by 8.6% (OR 0.914; 95% CI 0.868–0.962), each 1 cm/s increase in gait speed std. (DT) decreased the risk of disease by 20.8% (OR 0.792; 95% CI 0.665–0.994), each 1 cm increase in stride length (DT) decreased the risk of disease by 6.2% (OR 0.938; 95% CI 0.903–0.974), and each 1 s increase in turn duration (DT) increased the risk of disease by 6.902-fold (OR 7.907; 95% CI 2.401–26.039). From this, more significant gait characteristics were found in dual-task condition.

**Table 4:** Logistics regression analysis of single-task gait.

Variable	OR^1^	Value of *p*	95%CI	OR^2^	Value of *p*	95%CI
Gait speed (cm/s)	0.949	0.002	0.918–0.981	0.922	0.001	0.881–0.965
Stride length (cm)	0.955	0.001	0.929–0.981	0.939	0.001	0.906–0.972

OR^1^, un-adjusted for confounding factors; OR^2^, adjusted by gender, BMI and Osteoporotic.

**Table 5:** Logistics regression analysis of dual-task gait.

Variable	OR^1^	Value of *p*	95%CI	OR^2^	Value of *p*	95%CI
Gait Speed (cm/s) [DT]	0.940	0.003	0.902–0.978	0.914	0.001	0.868–0.962
Gait Speed std. (cm/s) [DT]	0.856	0.031	0.744–0.985	0.792	0.009	0.665–0.994
Stride Length (cm) [DT]	0.952	0.002	0.923–0.981	0.938	0.001	0.903–0.974
Turn Duration (s) [DT]	4.881	0.001	1.896–12.563	7.907	0.001	2.401–26.039

OR^1^, un-adjusted for confounding factors; OR^2^, adjusted by gender, BMI and Osteoporotic.

### 3.4. ROC curve

**Figure 2:**
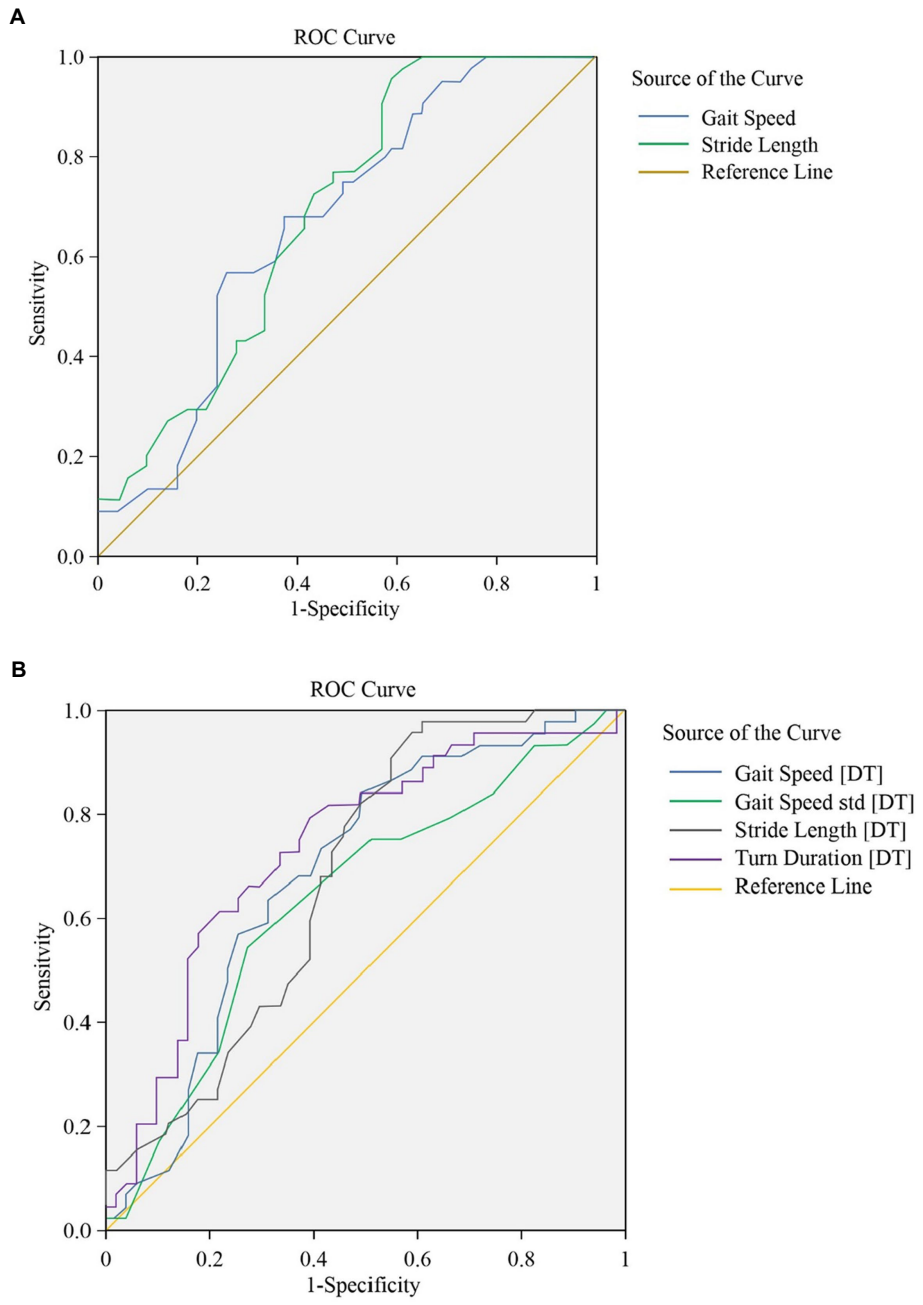
**(A)** The ROC for gait parameters (single task walking). Global performance of gait parameters under single-task walking for identification of frailty with sarcopenia. **(B)** The ROC for gait parameters (dual task walking). Global performance of gait parameters under dual-task walking for identification of frailty with sarcopenia.

**Figure 3:**
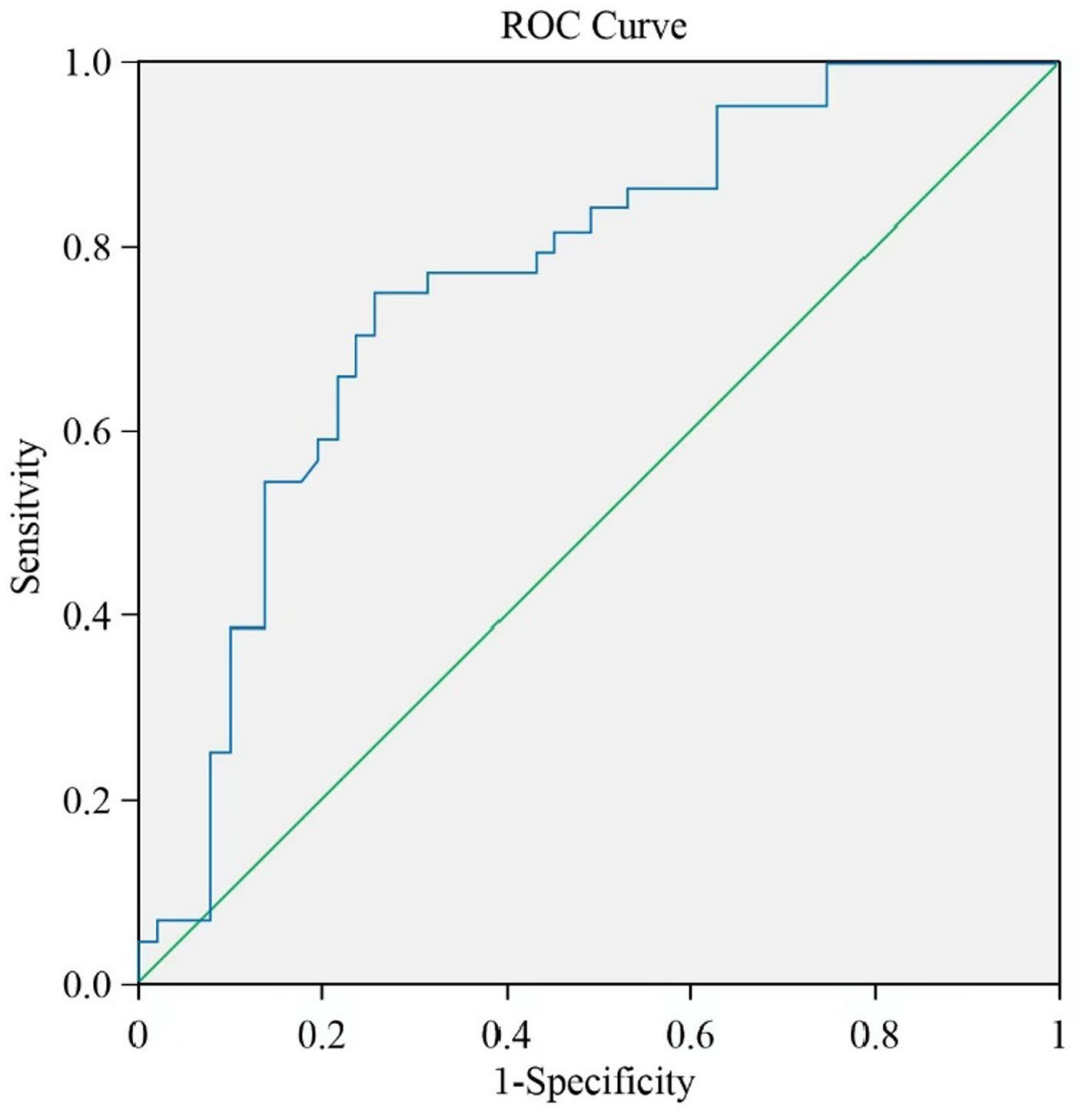
The ROC for a new gait model. A new model (gait speed [DT] + turn duration [DT]) noticeably improved AUC.

Figures 2A,B present Receiver Operating Curves (ROC) for gait parameters (single- and dual-task test) and sarcopenia in frail elderly. The parameters with a higher global performance were stride length 0.689 (0.584–0.795; *p* = 0.002), sensitivity (97.7%) and specificity (39.2%), turn duration (DT) 0.736 (0.633–0.838; *p* < 0.001) sensitivity (79.5%) and specificity (60.8%), and gait speed (DT) 0.688 (0.58–0.796; *p* = 0.002), sensitivity (84.1%) and specificity (51.0%). In general, the prediction abilities of dual-task gait parameters were better and more balanced than those of single-task gait parameters, see Table 6. We tried to combine these gait parameters together, and found the new model consisted of gait speed (DT) and turn duration (DT) (see Figure 3), AUC 0.763 (0.666–0.860; *p* < 0.001), sensitivity (75.0%) and specificity (74.5%), which showed a higher predictive performance, see Table 7.

**Table 6:** ROC for gait parameters and sarcopenia in frail elderly.

Variable	Sensitivity	Specificity	AUC	Value of *p*	95%CI	
					Lower bound	Upper bound
Single-task gait						
Gait Speed (cm/s)	56.8%	74.5%	0.673	0.004	0.565	0.781
Stride Length (cm)	97.7%	39.2%	0.689	0.002	0.584	0.795
Dual-task gait						
Gait Speed (cm/s) [DT]	84.1%	51.0%	0.688	0.002	0.58	0.796
Gait Speed std. (cm/s) [DT]	54.5%	72.5%	0.637	0.022	0.525	0.75
Stride Length (cm) [DT]	97.7%	39.2%	0.678	0.003	0.57	0.785
Turn Duration (s) [DT]	79.5%	60.8%	0.736	<0.001	0.633	0.838

**Table 7:** ROC for the new model (gait speed [DT] + turn duration [DT]).

Variable	Sensitivity	Specificity	AUC	Value of *p*	95%CI	
					Lower bound	Upper bound
Gait Speed [DT] + Turn Duration [DT]	75.0%	74.5%	0.763	<0.001	0.666	0.860

We also did a correlation analysis, turn duration (DT), gait speed (DT), and stride length (DT) were significantly correlated with the SMI and FREID scores, which verified that correlation also existed between turn duration (DT)/Gait speed (DT) and diagnostic criteria both for frailty and sarcopenia, see Table 8.

**Table 8:** Association between gait parameters and other study variables.

Variable	SMI	Grip strength	FREID scores
Gait Speed (cm/s) [DT]			
Correlation coefficient	0.460	0.092	−0.390
Value of *p*	<0.001	0.375	<0.001
Gait Speed std. (cm/s) [DT]			
Correlation coefficient	0.011	−0.029	0.006
Value of *p*	0.915	0.781	0.958
Stride Length (cm) [DT]			
Correlation coefficient	0.436	0.127	−0.383
Value of *p*	<0.001	0.211	<0.001
Turns Duration (s) [DT]			
Correlation coefficient	−0.374	−0.190	0.368
Value of *p*	<0.001	0.066	<0.001

## 4. Discussion

To our knowledge, this is the first time that gait analysis has been used for the identification of sarcopenia in the frail population. Compared with the non-sarcopenic participants, frail older adults with sarcopenia showed significantly worse gait performance. The most important finding of this study was the role of gait performance under dual-task conditions in identification of sarcopenia among frail elders, not only single-task gait performance. Our data revealed a novel and surprising role of turn duration under dual-task walking, which was that the prediction performance of turn duration (DT) was good alone or in combination. The combined model of gait speed (DT)-turn duration (DT) yields relatively higher accuracy, while balancing both convenience and ease of access. In the stage of frailty with sarcopenia, reduction of gait speed and increase of turn duration under dual-task conditions could be the more salient model of gait for sarcopenia in frail elderly populations.

In this study, the sociodemographic and medical characteristics of subjects from the two groups showed that frail patients with sarcopenia were leaner and presented with a higher proportion of male, lower gait speed, lower muscle mass, a higher rate of osteoporosis, and a higher degree of frailty, and these findings were in accordance with previous studies (Reiss et al., 2019; Wu et al., 2021). This category of individuals could be the group with a higher risk of adverse events, especially falls. For this reason, early screening or identification is of great significance. However, most studies were specific to frailty or sarcopenia alone, studies on frail patients with sarcopenia remain very scarce. Thus, we performed this pioneering research among the frail elderly with sarcopenia, and we hope this study may promote the development of a new screening method and a new insight into the diagnosis in such individuals.

The dual-task gait testing can reveal the subtleties about the role of cognitive control in participants’ gait, and patients with cognitive impairment often suffer from gait disorders (Studenski et al., 2003; Montero-Odasso et al., 2009, 2012a). In this study, we found that there was no significant difference between the two groups in MoCA scores of patients with frailty and frailty combined with sarcopenia (18.157 ± 4.483 vs. 17.295 ± 6.021, *p* > 0.05), we can thus exclude the impact of differences in cognitive function on outcome. In this regard, we demonstrated that these two patient groups were comparable. However, the cognitive level of the two groups was generally low, which even reached the level of mild cognitive impairment (MCI; Allali and Verghese, 2017). The correlation between cognitive impairment and frailty has been previously demonstrated in many studies (Buchman et al., 2008; Mulero et al., 2011). Our results coincided with previous studies, indicating that both frail elderly with and without sarcopenia would develop some degree of decline in cognitive capacity. However, frail older adults with sarcopenia did not present more severe cognitive impairment.

Stride speed can be used as a simple and time-saving screening tool for frailty and sarcopenia. Because it is one of the major diagnostic criteria both for frailty and sarcopenia, which is more related to muscular dystrophy, functional independence, vitality, and weakness (Fried et al., 2001; Tanimoto et al., 2012; Middleton et al., 2015). Thus, our result is not surprising that gait speed is a good predictor of sarcopenia in frail elderly. Because of the convenience of pace measurement, we ignore other gait parameters and the specificity of patient’s gait changes in other gait modes. In our research, it was found that the features of dual-task gait in cognitive intervention were more obvious than those of single-task gait. At the same time, we found that gait speed exhibited higher predictive performance under the dual-task walking. To our surprise, turn duration in dual-task testing had a stronger predictive effect than gait speed on identifying frailty with sarcopenia, this result remained significant even after controlling for potential confounds. At the same time, the turning assessment has been confirmed to be a potential way to identify older adults at fall risk, older fallers took significantly longer turning time than non-fallers (Yeh et al., 2022). In addition, the latest research suggests that turn duration also plays an important role in distinguishing the Parkinson’s disease patients from normal, and predicting the future risk of fall in poststroke hemiplegic individuals (Vitorio et al., 2021; Zou et al., 2021), these categories of individuals at a higher risk of falls could be potential frail patients with sarcopenia. Our result also validated that turn duration in the dual-task condition was more relevant to muscle mass, muscle function, and severity of frailty (SMI, FREID scores, and grip strength), compared with other dual-task gait parameters.

Using low-cost and convenient gait analysis techniques to determine sarcopenia is a promising prediction method in frail elderly populations (Mitsiopoulos et al., 1998; Levine et al., 2000; Beaudart et al., 2016). In this study, compared with other gait parameters, gait speed (DT) and turn duration (DT) displayed stronger predictive power. Compared to other indicators, these two indicators are not dependent on sophisticated gait assessment devices and trained professionals. These two fundamental measures can be easily accessed by a smartphone device with inertial measurement unit. Moreover, validity and reliability of mobile phone-based gait assessments for the elderly were demonstrated in recent studies (Serra-Añó et al., 2020; Silsupadol et al., 2020; Rashid et al., 2021). The addition of turn duration (DT) can increase the power to identify sarcopenia in frail elderly over the use of gait speed (DT) alone (increasing AUC from 0.68 to 0.76). Therefore, we believe that the combination of gait speed and turn duration under dual-task conditions could be a convenient and effective digital biomarker of sarcopenia in the frail older adults and will be suitable for application in primary health institutions.

A major strength in this study is the first detailed assessment of gait for sarcopenic patients in frail elderly, using the currently popular wearable platform (APDM’s Mobility Lab). The main benefit of using wearable sensors is that we can easily analyze detailed gait metrics (Mancini et al., 2011). Gait parameters were assessed not only in their normal walking pattern, but also under dual-task conditions. Additionally, this study assessed the cognitive functions of patients to understand changes across cognitive in the frailty with sarcopenia. Although the number of patients included in each group was modest, we did substantial work to screen residents in care homes for frailty. The disease may be often overlooked and requires a series of detailed assessments including grip strength, walking speed, and cognitive function, among others. Thus, we believe that these data are of high quality.

The major limitation of this study is its cross-sectional design. Although it is obvious that associations exist between gait changes and sarcopenia in patients with frailty, we cannot ascertain the temporal order. Although global cognitive function of the two groups did not hold obvious differences in this study, the tight relationship between brain network function and changes in gait has been conclusively demonstrated (Montero-Odasso et al., 2012b, 2015; Lo et al., 2017). It will be important for future studies to investigate further to understand how neural networks take part in the regulation process of gait changes, which would help to provide a better understanding of the mechanism of worse gait performance in frail and sarcopenic patients respectively, for a wider application of the gait-derived digital metrics.

## 5. Conclusion

This study shows that turn duration under dual-task is a better predictive marker for sarcopenia in frail older people compared to other gait parameters, and the dual-task walking is more effective than single-task walking in identifying frailty with sarcopenia. In addition to the gait speed, adding the gait index of turn duration can improve the ability to identify sarcopenia. We believe that our research results can provide more convenient and effective ideas for the future technical design of biomarkers for screening sarcopenia in the frail elderly, encourage future research to use gait indicators to detect the incidence of frailty with sarcopenia, and identify individuals at high risk of falls. Future studies are also recommended to use of gait indicators to promote timely intervention and evaluation of treatment results.

## Data availability statement

The raw data supporting the conclusions of this article will be made available by the authors, without undue reservation.

## Ethics statement

The studies involving human participants were reviewed and approved by the First Affiliated Hospital of Soochow University. The patients/participants provided their written informed consent to participate in this study. Written informed consent was obtained from the individual(s) for the publication of any potentially identifiable images or data included in this article.

## Author contributions

YW and HT designed this study. ZZ, YH, JW, and HS collected the patients’ data. ZZ, YH, JW, HS, HT, and YW participated in writing the article and gave final agreement to be accountable for all aspects of the work in ensuring that questions related to the accuracy or integrity of any part of the work were appropriately investigated and resolved. All authors contributed to the article and approved the submitted version.

## Funding

This work was partially supported by the Jiangsu Province Cadre Health Project (grant no. BJ19008). The leader project of clinical technology application research of Jiangsu elderly health research project (Project LR2021011).

## Conflict of interest

The authors declare that the research was conducted in the absence of any commercial or financial relationships that could be construed as a potential conflict of interest

## Publisher’s note

All claims expressed in this article are solely those of the authors and do not necessarily represent those of their affiliated organizations, or those of the publisher, the editors and the reviewers. Any product that may be evaluated in this article, or claim that may be made by its manufacturer, is not guaranteed or endorsed by the publisher.

## References

[ref1] Allali G. Verghese J. (2017). Management of gait changes and fall risk in MCI and dementia. Curr. Treat. Options Neurol. 19:29. doi: 10.1007/s11940-017-0466-1, PMID: 28733759

[ref2] AlMohaisen N. Gittins M. Todd C. Burden S. (2022). What is the overlap between malnutrition, frailty and sarcopenia in the older population? Study protocol for cross-sectional study using UK biobank. PLoS One 17:e0278371. doi: 10.1371/journal.pone.0278371, PMID: 36472992 PMC9725160

[ref3] Beaudart C. McCloskey E. Bruyère O. Cesari M. Rolland Y. Rizzoli R.. (2016). Sarcopenia in daily practice: assessment and management. BMC Geriatr. 16:170. doi: 10.1186/s12877-016-0349-4, PMID: 27716195 PMC5052976

[ref4] Binotto M. A. Lenardt M. H. Rodríguez-Martínez M. D. C. (2018). Physical frailty and gait speed in community elderly: a systematic review. Rev. Esc. Enferm. U.S.P. 52:e03392. doi: 10.1590/s1980-220x2017028703392, PMID: 30570081

[ref5] Brummel N. E. Bell S. P. Girard T. D. Pandharipande P. P. Jackson J. C. Morandi A.. (2017). Frailty and subsequent disability and mortality among patients with critical illness. Am. J. Respir. Crit. Care Med. 196, 64–72. doi: 10.1164/rccm.201605-0939OC, PMID: 27922747 PMC5519959

[ref6] Buchman A. S. Schneider J. A. Leurgans S. Bennett D. A. (2008). Physical frailty in older persons is associated with Alzheimer disease pathology. Neurology 71, 499–504. doi: 10.1212/01.wnl.0000324864.81179.6a, PMID: 18695161 PMC2676981

[ref7] Carmeli E. (2017). Frailty and primary sarcopenia: a review. Adv. Exp. Med. Biol. 1020, 53–68. doi: 10.1007/5584_2017_18 28382607

[ref8] Cederholm T. (2015). Overlaps between frailty and sarcopenia definitions. Nestle Nutr. Inst. Workshop Ser. 83, 65–69. doi: 10.1159/000382063, PMID: 26484770

[ref9] Chen L. K. Liu L. K. Woo J. Assantachai P. Auyeung T. W. Bahyah K. S.. (2014). Sarcopenia in Asia: consensus report of the Asian working Group for Sarcopenia. J. Am. Med. Dir. Assoc. 15, 95–101. doi: 10.1016/j.jamda.2013.11.025, PMID: 24461239

[ref10] Cruz-Jentoft A. J. Baeyens J. P. Bauer J. M. Boirie Y. Cederholm T. Landi F.. (2010). Sarcopenia: European consensus on definition and diagnosis: report of the European working group on sarcopenia in older people. Age Ageing 39, 412–423. doi: 10.1093/ageing/afq034, PMID: 20392703 PMC2886201

[ref11] Faxén-Irving G. Luiking Y. Grönstedt H. Franzén E. Seiger Å. Vikström S.. (2021). Do malnutrition, sarcopenia and frailty overlap in nursing-home residents? J. Frailty Aging 10, 17–21. doi: 10.14283/jfa.2020.45, PMID: 33331617

[ref12] Fried L. P. Tangen C. M. Walston J. Newman A. B. Hirsch C. Gottdiener J.. (2001). Frailty in older adults: evidence for a phenotype. J. Gerontol. A Biol. Sci. Med. Sci. 56, M146–M157. doi: 10.1093/gerona/56.3.m146 11253156

[ref13] Guedes R. C. Dias R. C. Pereira L. S. Silva S. L. Lustosa L. P. Dias J. M. (2014). Influence of dual task and frailty on gait parameters of older community-dwelling individuals. Braz. J. Phys. Ther. 18, 445–452. doi: 10.1590/bjpt-rbf.2014.0034, PMID: 25372007 PMC4228630

[ref14] Inoue A. Kuzuya M. Cheng X. (2018). Aging-related frailty and sarcopenia. Frailty-sarcopenia and biomarker. Clin. Calcium 28, 1191–1200, PMID: . 30146504

[ref15] Levine J. A. Abboud L. Barry M. Reed J. E. Sheedy P. F. Jensen M. D. (2000). Measuring leg muscle and fat mass in humans: comparison of CT and dual-energy X-ray absorptiometry. J. Appl. Physiol. (1985) 88, 452–456. doi: 10.1152/jappl.2000.88.2.452, PMID: 10658010

[ref16] Ligthart-Melis G. C. Luiking Y. C. Kakourou A. Cederholm T. Maier A. B. de van der Schueren M. A. E. (2020). Frailty, sarcopenia, and malnutrition frequently (co-)occur in hospitalized older adults: a systematic review and meta-analysis. J. Am. Med. Dir. Assoc. 21, 1216–1228. doi: 10.1016/j.jamda.2020.03.006, PMID: 32327302

[ref17] Liu D. Fan Y. B. Tao X. H. Pan W. L. Wu Y. X. Wang X. H.. (2021). Mitochondrial quality control in sarcopenia: updated overview of mechanisms and interventions. Aging Dis. 12, 2016–2030. doi: 10.14336/ad.2021.0427, PMID: 34881083 PMC8612607

[ref18] Lo O. Y. Halko M. A. Zhou J. Harrison R. Lipsitz L. A. Manor B. (2017). Gait speed and gait variability are associated with different functional brain networks. Front. Aging Neurosci. 9:390. doi: 10.3389/fnagi.2017.00390, PMID: 29249961 PMC5715372

[ref19] Mancini M. King L. Salarian A. Holmstrom L. McNames J. Horak F. B. (2011). Mobility lab to assess balance and gait with synchronized body-worn sensors. J. Bioeng. Biomed. Sci. Suppl. Suppl_1:007. doi: 10.4172/2155-9538.S1-007, PMID: 24955286 PMC4062543

[ref20] Marques A. Queirós C. (2018). “Frailty, sarcopenia and falls” in Fragility fracture nursing: holistic care and management of the orthogeriatric patient. eds. Hertz K. Santy-Tomlinson J. (Cham (CH): Springer), 15–26. Copyright 2018, The Editor(s)(if applicable) and the Author(s) 31314236

[ref21] Middleton A. Fritz S. L. Lusardi M. (2015). Walking speed: the functional vital sign. J. Aging Phys. Act. 23, 314–322. doi: 10.1123/japa.2013-0236, PMID: 24812254 PMC4254896

[ref22] Mijnarends D. M. Schols J. M. Meijers J. M. Tan F. E. Verlaan S. Luiking Y. C.. (2015). Instruments to assess sarcopenia and physical frailty in older people living in a community (care) setting: similarities and discrepancies. J. Am. Med. Dir. Assoc. 16, 301–308. doi: 10.1016/j.jamda.2014.11.011, PMID: 25530211

[ref23] Mitsiopoulos N. Baumgartner R. N. Heymsfield S. B. Lyons W. Gallagher D. Ross R. (1998). Cadaver validation of skeletal muscle measurement by magnetic resonance imaging and computerized tomography. J. Appl. Physiol. (1985) 85, 115–122. doi: 10.1152/jappl.1998.85.1.115, PMID: 9655763

[ref24] Montero-Odasso M. Bherer L. Studenski S. Gopaul K. Oteng-Amoako A. Woolmore-Goodwin S.. (2015). Mobility and cognition in seniors. Report from the 2008 Institute of Aging (CIHR) mobility and cognition workshop. Can. Geriatr. J. 18, 159–167. doi: 10.5770/cgj.18.188, PMID: 26495050 PMC4597816

[ref25] Montero-Odasso M. Casas A. Hansen K. T. Bilski P. Gutmanis I. Wells J. L.. (2009). Quantitative gait analysis under dual-task in older people with mild cognitive impairment: a reliability study. J. Neuroeng. Rehabil. 6:35. doi: 10.1186/1743-0003-6-35, PMID: 19772593 PMC2754991

[ref26] Montero-Odasso M. Muir S. W. Speechley M. (2012a). Dual-task complexity affects gait in people with mild cognitive impairment: the interplay between gait variability, dual tasking, and risk of falls. Arch. Phys. Med. Rehabil. 93, 293–299. doi: 10.1016/j.apmr.2011.08.026, PMID: 22289240

[ref27] Montero-Odasso M. Schapira M. Soriano E. R. Varela M. Kaplan R. Camera L. A.. (2005). Gait velocity as a single predictor of adverse events in healthy seniors aged 75 years and older. J. Gerontol. A Biol. Sci. Med. Sci. 60, 1304–1309. doi: 10.1093/gerona/60.10.1304, PMID: 16282564

[ref28] Montero-Odasso M. Verghese J. Beauchet O. Hausdorff J. M. (2012b). Gait and cognition: a complementary approach to understanding brain function and the risk of falling. J. Am. Geriatr. Soc. 60, 2127–2136. doi: 10.1111/j.1532-5415.2012.04209.x, PMID: 23110433 PMC3498517

[ref29] MuleroJ.ZafrillaP.Martinez-CachaA. (2011). Oxidative stress, frailty and cognitive decline. J. Nutr. Health Aging 15, 756–760. doi: 10.1007/s12603-011-0130-522089224

[ref30] Petermann-Rocha F. Gray S. R. Pell J. P. Ho F. K. Celis-Morales C. (2021). The joint association of sarcopenia and frailty with incidence and mortality health outcomes: a prospective study. Clin. Nutr. 40, 2427–2434. doi: 10.1016/j.clnu.2020.10.044, PMID: 33189425

[ref31] Pfortmueller C. A. Lindner G. Exadaktylos A. K. (2014). Reducing fall risk in the elderly: risk factors and fall prevention, a systematic review. Minerva Med. 105, 275–281, PMID: . 24867188

[ref32] Rashid U. Barbado D. Olsen S. Alder G. Elvira J. L. L. Lord S.. (2021). Validity and reliability of a smartphone app for gait and balance assessment. Sensors (Basel) 22:124. doi: 10.3390/s22010124, PMID: 35009667 PMC8747233

[ref33] Reiss J. Iglseder B. Alzner R. Mayr-Pirker B. Pirich C. Kässmann H.. (2019). Sarcopenia and osteoporosis are interrelated in geriatric inpatients. Z. Gerontol. Geriatr. 52, 688–693. doi: 10.1007/s00391-019-01553-z, PMID: 31049683 PMC6817738

[ref34] Roberts S. Collins P. Rattray M. (2021). Identifying and managing malnutrition, frailty and sarcopenia in the community: a narrative review. Nutrients 13:2316. doi: 10.3390/nu13072316, PMID: 34371823 PMC8308465

[ref35] Rothman M. D. Leo-Summers L. Gill T. M. (2008). Prognostic significance of potential frailty criteria. J. Am. Geriatr. Soc. 56, 2211–2216. doi: 10.1111/j.1532-5415.2008.02008.x, PMID: 19093920 PMC2782664

[ref36] Sáez de Asteasu M. L. Martínez-Velilla N. Zambom-Ferraresi F. Casas-Herrero Á. Ramirez-Vélez R. Izquierdo M. (2019). Role of muscle power output as a mediator between gait variability and gait velocity in hospitalized older adults. Exp. Gerontol. 124:110631. doi: 10.1016/j.exger.2019.110631, PMID: 31201920

[ref37] Serra-Añó P. Pedrero-Sánchez J. F. Inglés M. Aguilar-Rodríguez M. Vargas-Villanueva I. López-Pascual J. (2020). Assessment of functional activities in individuals with Parkinson's disease using a simple and reliable smartphone-based procedure. Int. J. Environ. Res. Public Health 17:4123. doi: 10.3390/ijerph17114123, PMID: 32527031 PMC7312659

[ref38] Silsupadol P. Prupetkaew P. Kamnardsiri T. Lugade V. (2020). Smartphone-based assessment of gait during straight walking, turning, and walking speed modulation in laboratory and free-living environments. IEEE J. Biomed. Health Inform. 24, 1188–1195. doi: 10.1109/jbhi.2019.2930091, PMID: 31329138

[ref39] Studenski S. Perera S. Wallace D. Chandler J. M. Duncan P. W. Rooney E.. (2003). Physical performance measures in the clinical setting. J. Am. Geriatr. Soc. 51, 314–322. doi: 10.1046/j.1532-5415.2003.51104.x 12588574

[ref40] Tanimoto Y. Watanabe M. Sun W. Sugiura Y. Tsuda Y. Kimura M.. (2012). Association between sarcopenia and higher-level functional capacity in daily living in community-dwelling elderly subjects in Japan. Arch. Gerontol. Geriatr. 55, e9–e13. doi: 10.1016/j.archger.2012.06.015, PMID: 22795189

[ref41] Thiede R. Toosizadeh N. Mills J. L. Zaky M. Mohler J. Najafi B. (2016). Gait and balance assessments as early indicators of frailty in patients with known peripheral artery disease. Clin. Biomech. (Bristol, Avon) 32, 1–7. doi: 10.1016/j.clinbiomech.2015.12.002, PMID: 26775227 PMC4779419

[ref42] Thompson M. Q. Yu S. Tucker G. R. Adams R. J. Cesari M. Theou O.. (2021). Frailty and sarcopenia in combination are more predictive of mortality than either condition alone. Maturitas 144, 102–107. doi: 10.1016/j.maturitas.2020.11.009, PMID: 33358201

[ref43] Verghese J. Holtzer R. Lipton R. B. Wang C. (2012). Mobility stress test approach to predicting frailty, disability, and mortality in high-functioning older adults. J. Am. Geriatr. Soc. 60, 1901–1905. doi: 10.1111/j.1532-5415.2012.04145.x, PMID: 23002714 PMC3470773

[ref44] Verlinden V. J. van der Geest J. N. Hoogendam Y. Y. Hofman A. Breteler M. M. Ikram M. A. (2013). Gait patterns in a community-dwelling population aged 50 years and older. Gait Posture 37, 500–505. doi: 10.1016/j.gaitpost.2012.09.005, PMID: 23018028

[ref45] Vitorio R. Hasegawa N. Carlson-Kuhta P. Nutt J. G. Horak F. B. Mancini M.. (2021). Dual-task costs of quantitative gait parameters while walking and turning in people with Parkinson's disease: beyond gait speed. J. Parkinsons Dis. 11, 653–664. doi: 10.3233/jpd-202289, PMID: 33386812

[ref46] Wewerka G. Wewerka G. Iglseder B. (2015). Measuring gait velocity in the elderly with a gait analysis system and a 10-meter walk test. A comparison. Z. Gerontol. Geriatr. 48, 29–34. doi: 10.1007/s00391-013-0569-6, PMID: 24292516

[ref47] Wu L. C. Kao H. H. Chen H. J. Huang P. F. (2021). Preliminary screening for sarcopenia and related risk factors among the elderly. Medicine (Baltimore) 100:e25946. doi: 10.1097/md.0000000000025946, PMID: 34106666 PMC8133124

[ref48] Yeh T. T. Liang P. J. Lee S. C. (2022). Differences in walking-to-turning characteristics between older adult fallers and nonfallers: a prospective and observational study using wearable inertial sensors. Int. J. Rehabil. Res. 45, 53–57. doi: 10.1097/mrr.0000000000000511, PMID: 34860731

[ref49] Yu J. Li J. Huang X. (2012). The Beijing version of the Montreal cognitive assessment as a brief screening tool for mild cognitive impairment: a community-based study. BMC Psychiatry 12:156. doi: 10.1186/1471-244x-12-156, PMID: 23009126 PMC3499377

[ref50] Zou T. E. Liang P. J. Lee S. C. (2021). Turning duration and steps predict future falls in poststroke hemiplegic individuals: a preliminary cohort study. Top. Stroke Rehabil. 28, 33–41. doi: 10.1080/10749357.2020.1760644, PMID: 32397952

